# The impact of less restrictive quarantine for exposed COVID-19 patients in inpatient psychiatric settings: a cohort analysis

**DOI:** 10.1017/ash.2025.10041

**Published:** 2025-06-23

**Authors:** Krishnendu Mangal, Mikaela Kluver, Graham M. Snyder, Janina-Marie Huss

**Affiliations:** 1 Department of Infection Prevention and Control, UPMC Senior Communities, Pittsburgh, PA, USA; 2 Department of Infection Prevention and Control, UPMC Western Psychiatric Hospital, Pittsburgh, PA, USA; 3 Division of Infectious Diseases, University of Pittsburgh School of Medicine, Pittsburgh, PA, USA; 4 Department of Infection Prevention and Control, UPMC Presbyterian, Pittsburgh, PA, USA; 5 System Quality, WVU Medicine, Morgantown, WV, USA

## Abstract

**Objective:**

To quantify the impact of a changing cohort strategy on COVID-19 transmission in an acute inpatient behavioral health facility.

**Design:**

Cohort study.

**Patients:**

Behavioral health inpatients exposed to COVID-19.

**Interventions:**

This cohort project compared COVID-19 conversion rates during two periods. In the first period (July 2020–April 2022), exposed patients (regardless of vaccination status) were cohorted separately from unexposed individuals. In the second period (May 2022–September 2022), exposed vaccinated patients remained with unexposed patients. COVID-19 conversion was identified through post-exposure asymptomatic testing or test-confirmed symptom development, with rates quantified per all admissions, per 10,000 patient days at risk, and per patient-specific exposure.

**Results:**

The 27-month project included 11,761 admissions and 164,762 patient days of care. The proportion of patients up-to-date on COVID-19 vaccination at admission and discharge ranged from 11%–19%. The second period showed an increased risk of SARS-CoV-2 conversion per admission (1.87% vs 0.36%, *P* < 0.001) and per 1,000 patient-days at risk (1.44 vs 0.27 conversions per 1,000 patient days, *P* < 0.001), but not per exposure (3.44% vs 3.13%, *P* = 0.68).

**Conclusions:**

Reducing the population of patients cohorted after a SARS-COV-2 exposure is associated with increased risk of SARS-COV-2 transmission in inpatient psychiatric settings.

## Introduction

Transmission of SARS-CoV-2, the causative agent of coronavirus disease 2019 (COVID-19), has been demonstrated in acute care settings including in psychiatric facilities.^1^ Rates of transmission ranging from 30% to 40% have been observed among patients admitted to shared hospital rooms, despite infrequent instances of prolonged face-to-face contact between patients when compared to household settings.^1,2^ The design and delivery of care in inpatient psychiatric facilities pose particular challenges in preventing SARS-CoV-2 exposure among patients, staff, and visitors. Patients residing in these facilities often share bedrooms, participate in group therapies, dine in communal cafeterias, and have difficulties with personal hygiene, increasing their risk of infection.^3,4^ Previously described strategies within inpatient psychiatric facilities have included visitor restrictions, patient screening for asymptomatic carriage, suspended group activities, and cohorting of COVID-19 exposed and contagious patients.^5–7^ Optimal approaches to preventing SARS-CoV-2 transmission in inpatient psychiatric settings are not established.^8^

Individuals receiving psychiatric or mental health care are among the groups that are highly susceptible to the impacts of the COVID-19 pandemic, due to an increased risk of exposure to the virus and complications associated with medical comorbidities.^1^ As the pandemic continues, the protective measures implemented in healthcare settings to mitigate the spread of the virus have evolved. As of September 23rd, 2022, the CDC no longer recommended using vaccination status to inform source control, post-exposure recommendation, and screening tests.^9^ While no psychiatric setting-specific guidance is promulgated by the CDC, the Pennsylvania Department of Health has issued guidance “specific for long-term care facilities (LTCF) but may also be applicable to other congregate and residential settings;” in the absence of behavioral health-specific guidance, this guidance was accepted by the healthcare community as applying to inpatient psychiatric settings. One advisory update, Pennsylvania Health Alert Network (PA HAN) 627, “Response to an Outbreak and Residents with exposure to COVID-19 for Long-term Care Facilities,” released on February 15^th^, 2022, provided an opportunity to compare different transmission mitigation strategies in psychiatric settings.^10^ Prior to PA HAN 627, the infection prevention and control measures were not dependent on vaccination status, and all patients exposed to SARS-CoV-2 were cohorted separately from unexposed and non-contagious individuals. PA HAN 627 guidance indicated that patients exposed to SARS-CoV-2 who have received all the recommended COVID-19 vaccines and those who have recuperated from the illness within the preceding 90 days no longer required cohorting and could continue to receive congregate care with unexposed and non-contagious individuals.^10^ The extent to which vaccination, combined with symptom monitoring, post-exposure asymptomatic screening, and universal precautions including masking, is sufficient to prevent transmission in inpatient psychiatric settings is not known.

The aim of this investigation was to determine the relative risk of SARS-CoV-2 transmission in inpatient psychiatric settings when comparing a strategy of vaccination status-independent cohorting of exposed individuals versus vaccine status-dependent continued congregate care.

## Methods

### Project setting and population

This project took place in a facility in western Pennsylvania that serves as a regional center for psychiatric inpatient and ambulatory care, research, and medical education. The hospital comprises 263 inpatient beds in 12 condition-defined units including general child and adolescent unit, general adult services, integrated health and ageing program, comprehensive recovery services, dual diagnosis unit, inpatient center for eating disorders, adolescent bipolar assessment unit, center for autism and developmental disorders units, transitional recovery, and comprehensive recovery services. This project was approved as a non-research quality improvement investigation by the UPMC Quality Review Committee.

This cohort project included all patients admitted for at least one calendar day from July 1^st^, 2020, through September 30^th^, 2022. Patients were included if they were inpatient in one of ten designated units (A through J). Since psychiatric emergency services and outpatient visits did not always consist of at least one inpatient day, patients in those units were excluded from the project.

During the study period, the following COVID-19 pandemic practices did not change: Testing methodology, asymptomatic screening of patients, admission process and clinical unit designations, employee health illness policy^11^, visitor policy, and use of personal protective equipment.

### Outcomes

The primary project outcome was the SARS-CoV-2 conversion rate, quantified among patients at risk for infection and exposed to a SARS-CoV-2 contagious individual. A conversion was defined as a positive test for SARS-CoV-2 (asymptomatic or symptomatic) within 10 days of their last known exposure to a contagious individual. A patient was considered exposed if they were in the same unit or communal areas of the hospital for any amount of time on a calendar day as another patient in the contagious period of SARS-CoV-2 infection. A patient is considered unexposed if they were not cared for in a shared unit or communal area with a contagious patient or were themselves an exposure source or after becoming SARS-CoV-2 contagious after exposure. Susceptible individuals were all patients without a positive test result within the 90 days prior to exposure. Patient days at risk were defined as inpatient admission days for which a susceptible patient had not had a positive test for SARS-CoV-2 in the preceding 90 days. The conversion rate was calculated using the conversion outcome with three different denominator-defined rates: (1) per all hospital admissions, (2) per susceptible individuals who were exposed, (3) and per patient days at risk for infection.

We compared the SARS-COV-2 conversion rate in two periods and patient populations. First, we compared the conversion rates during the project period July 2020 through April 2022 (“project period 1”), during which time exposed patients (of any vaccination status) were cohorted in a location different than unexposed and non-contagious individuals, and the project period May 2022 through September 2022 (“project period 2”), a period where SARS-CoV-2 exposed vaccinated patients remained in care locations with unexposed patients.

### Data sources

The project team used quality surveillance software (TheraDoc, Premier Inc., Charlotte, NC) to generate lists of patients and admissions. SARS-CoV-2 testing, patient exposure to SARS-CoV-2 contagious patients, and the location of exposure and testing were derived from data sets used for routine hospital operations.

Patients with multiple admissions were only counted once for the description of demographics, where age and sex were used only once for each unique individual. In instances of multiple admissions, the average age of the patient was selected for analysis.

The Pennsylvania Statewide Immunization Information System (PA SIIS) was used to acquire the COVID-19 vaccination records of individuals who were inpatient during the project period, collecting data on the type of vaccination, date of the primary series vaccination, and any booster or bivalent shots received.^12^ Patients were considered up to date if they had received all of the recommended COVID-19 vaccine doses at the time of admission, based on the individual’s age, the type of vaccine they received, and the duration of time since the last dose.

We also characterized the findings from the epidemiological investigation of transmission within the facility, as performed by Infection Prevention & Control. An outbreak cluster was defined as patients with a positive test for SARS-CoV-2 with an epidemiologic linkage to one or more other patients. An epidemiologic linkage was defined as occupying shared space during the contagious window of the source and development of symptoms or positive test within the incubation period for the exposed individual. The number of outbreak clusters in each period, and the distribution size (median, interquartile range) of outbreak clusters in each period, were calculated as descriptive analyses.

To qualitatively assess the frequency of SARS-CoV-2 cases in the project population compared to the community, epidemiological curves were drawn for COVID-19-positive cases in Allegheny County, PA, where the facility is located, using the Western Pennsylvania Regional Data Center.^13^ We used all positive SARS-CoV-2 tests at the project facility to define an institution epidemiological curve.

### Statistical analysis

Microsoft Excel was utilized to categorize patients based on their age, gender, vaccination expiration dates in accordance with the PA HAN 627 vaccination criteria, and their up-to-date vaccination status deemed by their admission dates. Comparisons in conversion rates were performed using STATA (version 12.1). Comparisons of proportions of admissions and exposed individuals were calculated using a 2-sample test of proportions, with significance defined as p-value <0.05; comparisons in conversions per patient days calculated as an incidence rate ratio with 95% confidence interval and p-values calculated using the mid-p adjustment to exact p-values, with p-value <0.05 defined as significant.

## Results

**Figure 1.**
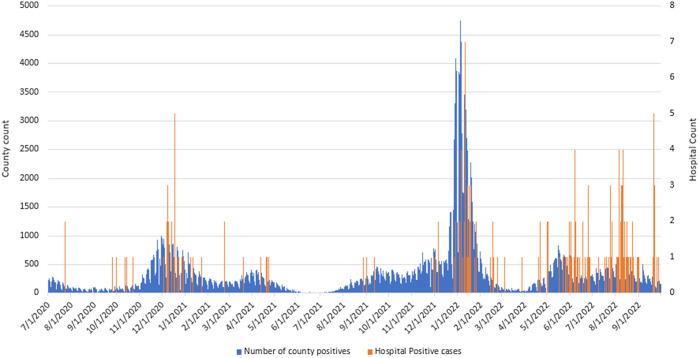
COVID-19 positive count for Allegheny County and project hospital between July 1st 2020 and September 30th 2022.

**Figure 2.**
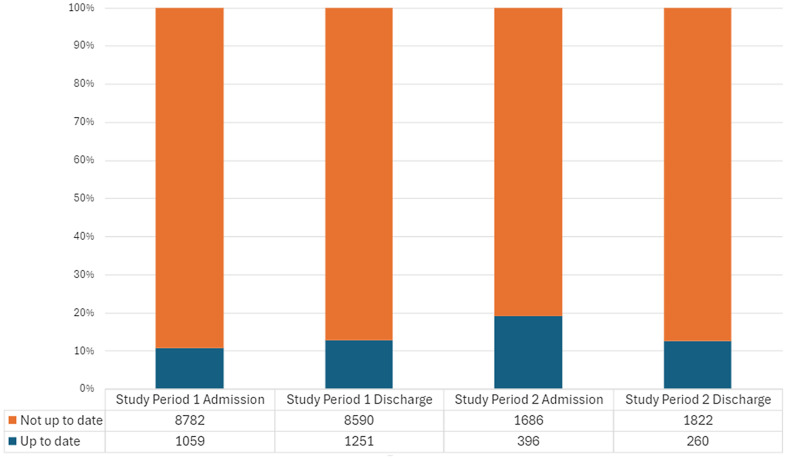
Vaccination status of patients admitted to the project hospital, at the time of admission and discharge, before and after change to COVID-19 exposure management.

During the entire 27-month project period, there were 11,761 admissions accounting for 164,762 patient days of care (Table 1). The median (range) age of the source population was 28.6 (4.6-110.0) years, and 49.0% of patient admissions were for male (one patient’s sex was recorded as “unknown”). The epidemiological curve for all positive testing at the project facility, and for the surrounding county, is shown in Figure 1. During project period 1, 11% of patients were up to date with COVID-19 vaccination at the time of admission, compared with 19% at the time of admission in project period 2 (Figure 2).

**Table 1.** Characteristics of patients admitted to an inpatient psychiatric hospital between July 2020 and September 2022, including COVID-19 exposures and conversions

Characteristics	Period 1	Period 2
Admissions	9,840	1,921
Patient days	137,732	27,030
Patient days at risk	132,031	25,012
Exposures	1,120	1,045
Index cases resulting in an exposure	91	79
Conversions following exposure	35	36
Conversion rate, all admissions (conversions/admissions), %	0.36%	1.87%
Conversion rate, exposed individuals (conversions/exposed), %	3.13%	3.44%
Conversion rate per 1,000 patient days at risk	0.27	1.44

In project period 1, there were 91 index cases of SARS-CoV-2 resulting in an exposure to other patients, and 79 index cases in project period 2. These exposures resulted in 35 SARS-CoV-2 conversions in project period 1 and 36 SARS-CoV-2 conversions in project period 2 (Table 1). The change in cohorting strategies from project period 1 to project period 2 was associated with a significant increase of 1.52% in the conversion rate per admission (*P* < 0.001) and a non-significant increase of 0.32% in the conversion rate per exposure (*P* = 0.68) (Table 1). Project period 2 was associated with a significantly higher conversion rate per 10,000 patient days at risk (incidence rate ratio, 5.43; 95% confidence interval 3.31-8.90; *P* < 0.001) (Table 1).

In the 22 months of project period 1, there were 19 epidemiologically defined clusters with a median size 2 (range, 2–7), and in 5 months of project period 2, there were 8 epidemiologically defined clusters with a median cluster size of 4 (range, 2–12).

## Discussion

In this 27-month cohort project of all admissions to an inpatient psychiatric hospital, we found that SARS-CoV-2 conversions were more likely in a period during which SARS-CoV-2 exposed vaccinated patients remained in care with unexposed patients, as compared to a period where all exposed patients were cohorted separate from unexposed and non-contagious individuals, when measured as conversions per admission (1.87% vs 0.36% of all admissions) and per 10,000 patient days at risk (1.44 vs 0.27 conversions per 10,000 days), but not per exposure (3.44% vs 3.13%). While an “expected” frequency is not feasible to calculate, two observations suggested an increased risk of transmission after the change in cohorting strategy. The frequency of clusters identified by Infection Prevention and Control increased from 19 clusters in 22 months (project period 1) to 8 clusters in 5 months (project period 2), with a numerically larger clusters size (median increased from 2 to 4); on ecological and qualitative analysis, the frequency of SARS-CoV-2 acquisition during the second project period exceeded what might be expected based on trends in regional public health-reported community prevalence. These findings suggest that while the likelihood of conversion following an exposure did not change between the two project periods, a change to a less constrictive and vaccination-dependent cohorting strategy was associated with a significant but modest increased risk of SARS-CoV-2 transmission.

From both a societal perspective and public health approach, our communities are incrementally relaxing precautions to prevent transmission of SARS-CoV-2.^14,15^ In healthcare settings, this change is occurring to reduce barriers to delivery of care, and this is true for the resource-constrained area of acute psychiatric care. A change in cohorting practice to be more permissive of transmission risk—such as the change evaluated in this investigation—may benefit patients from improving access to psychiatric care (where inpatient care is required) and patient experience but increase harm from transmissible diseases.^16,17^ The significant but relatively small absolute increased risk we observed may be informative to psychiatric healthcare organization leaders and policy makers in planning safe but maximally inclusive and effective care.

Our evaluation was not designed to experimentally test varying approaches to preventing SARS-CoV-2 transmission, including admission and/or periodic asymptomatic testing,^6–8^ universal masking,^18–20^ social distancing (eg, minimum of 6 feet),^21,22^ enhanced air circulation and/or purification,^23,24^ or other measures. However, the cost of measures beyond healthcare setting standards-of-care (eg, air circulation, evaluation, and management of symptomatic persons), including both monetary cost and as barriers to the patient experience of psychiatric care, likely now make them prohibitive to continue implementing. The findings in the context of the facility approaches during this investigation—universal masking, asymptomatic screening on admission, and healthcare-standard air circulation without enhancement—may not be generalizable to situations where measures have been de-escalated.

Our investigation is subject to additional limitations. We cannot account for full assessment of exposure and acquisition, nor control for all factors (eg, time since vaccination, quantitative degree of exposure, and temporal trends) that may influence the relationship between cohorting strategy and SARS-CoV-2 transmission. While other measures that may impact SARS-CoV-2 transmission did not change during the study period, we cannot quantify adherence to these measures. The observed relationship is an association and not necessarily causal. Additionally, transmission was inferred based on epidemiological findings and not routinely confirmed with genetic relatedness testing.^25^ The conversion rates we observed were lower than observed in outbreak reports and studies of household transmission, particularly given low COVID-19 vaccine uptake in this population. The difference may be attributable to case ascertainment, though post-exposure symptom monitoring, symptomatic testing, and asymptomatic screening reliably conducted while patients remained admitted following exposure. The lower rates in our project may be a “true” estimate—inpatient congregate psychiatric care may be more like similar rates reported for acute inpatient medical care than for household encounters due to factors like proximity, space, and air circulation.^26,27^ A future research agenda should include a more focused assessment of respiratory virus transmission risk in inpatient settings, including psychiatric care.

## Conclusion

In this cohort project, observing the change in SARS-CoV-2 conversion rate among inpatient psychiatric patients after reducing the post-exposure cohorted population from all exposed patients to only those who were unvaccinated, the rate of SARS-CoV-2 conversions per admission and per days at risk significantly increased by the conversion rate per exposure did not significantly change. A less restrictive cohorting strategy was associated with increased transmission risk at the population level. Ongoing studies in psychiatric settings should continue to quantify the risk of respiratory virus transmission, and refine estimates of protective effects of physical, environmental, and immunological (vaccination) measures to reduce nosocomial infections.
